# Corosolic acid alleviates rheumatoid arthritis by down regulation of the NF-κB/PI3K/AKT signaling pathway

**DOI:** 10.1038/s41598-026-46070-3

**Published:** 2026-03-28

**Authors:** Xian Jiang, Weixi Liu, Shanqi Xu, Xinling Ni, Weiding Cui, Zhicheng Yang, Xiaodong Chen, Liqun Wang, Ruiping Liu

**Affiliations:** 1 Department of Biochemical Engineering, College of Pharmaceutical Engineering and Life Science, Changzhou University, Changzhou, 213164 Jiangsu China; 2 Department of Orthopedic Surgery, Xinhua Hospital, Shanghai Jiao Tong University School of Medicine, Shanghai, 200092 China; 3 Department of Orthopaedics, The Affiliated Changzhou No. 2 People’s Hospital of Nanjing Medical University, Changzhou, 213003 China; 4 Nanjing Medical University, Nanjing, 210029 China; 5 Changzhou Medical Center, Nanjing Medical University, Changzhou, 213003 China

**Keywords:** Corosolic acid, Rheumatoid arthritis, Fibroblast like synovial cells, NF-κB, PI3K/AKT, Diseases, Drug discovery, Immunology, Medical research, Rheumatology

## Abstract

Rheumatoid arthritis (RA) is an autoimmune disease characterized by synovitis. Existing treatments for RA have serious side effects. Corosolic acid (CRA) is a natural pentacyclic triterpenoid compound with anti-inflammatory potential. However, the effect of CRA on inflammation, its possible mechanism of action, and potential therapeutic effect on RA are not well understood. This study explored the CRA mechanism of treatment action on RA by evaluating the NF-κB/PI3K/AKT signaling pathway in vitro and in vivo. In vitro, fibroblast like synovial cells (FLS) from collagen induced arthritis (CIA) rats were assessed. ELISA, western blot, and immunofluorescence techniques demonstrated CRA (6 µg/mL) to significantly inhibit FLS secretion of TNF-α, IL-1β, and IL-6, as well as downregulate the phosphorylation of NF-κB and PI3K/AKT pathways. Inhibition experiments demonstrated that NF-κB activation was upstream of PI3K/AKT activation. In vivo, after 21 days of oral administration of CRA (10 mg/kg) to CIA rats, the arthritis score, organ index, and serum inflammatory factor levels were significantly decreased. Histopathologic analysis and Micro-CT showed that CRA reduced synovial hyperplasia and bone erosion. These results indicated that CRA exerted anti-inflammatory effects by inhibition of the NF-κB/PI3K/AKT signaling pathway, providing a potential new treatment strategy for RA patients.

**Supplementary Information:**

The online version contains supplementary material available at 10.1038/s41598-026-46070-3.

## Introduction

Rheumatoid arthritis (RA) is a systemic autoimmune disease characterized by chronic synovitis and pannus formation that progressively invades and destroys articular cartilage and adjacent bone, ultimately leading to jointdeformity^1^. The global prevalence of RA is approximately 0.5–1.0%, and the disease is associated with substantial disability, imposing a significant burden on both patients and society^2^. Current pharmacologic management relies mainly on non-steroidal anti-inflammatory drugs (NSAIDs), disease-modifying antirheumatic drugs (DMARDs), and biologic agents. However, these therapies may cause adverse effects, including hepatotoxicity, nephrotoxicity, gastrointestinal ulceration, and cardiovascular complications^3,4^. Thus, there remains an urgent need to identify safer, more affordable, and effective therapeutic options for RA.

The synovium is a primary site of inflammation in RA. The inflamed synovial tissue contains FLS, macrophages, and other immune and stromal cell populations, which together shape the inflammatory microenvironment and influence synovial homeostasis^5^. Among these, FLS are key effectors driving synovial hyperplasia and persistent inflammation. Activated FLS continuously produce pro-inflammatory cytokines (e.g., TNF-α, IL-1β, and IL-6) and matrix metalloproteinases (MMPs), thereby promoting cartilage damage and joint destruction^6^. Importantly, RA-FLS exhibit apoptosis resistance and an aggressive, invasive phenotype, contributing to synovial lining expansion and pannus formation that adhere to and invades cartilage and bone, further accelerating joint injury^7^. Accordingly, targeting FLS activation and their production of inflammatory mediators represents a promising strategy for RA intervention.

Traditional Chinese medicine has long been used in the management of inflammatory disorders, including RA. Several natural products and herbal-derived compounds (e.g., galangin, sinomenine, and Tripterygium glycosides) have been reported to exhibit anti-inflammatory effects in experimental RA models and/or clinical contexts^8–10^. Among natural products, triterpenoids have attracted attention because of their anti-inflammatory and other biological activities^11,12^. Current RA-related triterpenoid research mainly involves three classes: pentacyclic triterpenes, tetracyclic and rearranged triterpenes, and triterpenic saponins^13^. Pentacyclic triterpenoids are one of them. Corosolic acid (CRA) is a type of pentacyclic triterpenoid. CRA is a pentacyclic triterpenoid found in loquat leaves (Eriobotrya japonica), Lagerstroemia species, and other plants. CRA has been reported to exert anti-inflammatory and antioxidant activities and to ameliorate diabetic kidney injury, reduce blood pressure, inhibit osteoclastogenesis, and attenuate osteolysis^14–17^.

A recent study reported that CRA alleviated osteoarthritis by activating autophagy through the phosphatidylinositol 3-kinase/protein kinase B/mammalian target of rapamycin (PI3K/AKT/mTOR) pathway, thereby reducing IL-1β–induced extracellular matrix degradation in rat chondrocytes^18^. Although RA and osteoarthritis differ substantially in etiology and immune mechanisms, both involve inflammatory components; therefore, CRA may have potential relevance to RA. Notably, PI3K/AKT signaling is aberrantly activated in RA synovial tissue and contributes to inflammatory mediator production^19^. In addition, nuclear factor kappa B (NF-κB) is a central transcription factor that promotes expression of numerous inflammatory mediators during RA, including TNF-α, IL-1β, and IL-6^20^. Because NF-κB and PI3K/AKT signaling are both implicated in RA pathogenesis, it is important to clarify whether CRA exerts anti-arthritic effects by modulating these pathways.

In this study, we investigated CRA as a potential therapeutic candidate for RA. Specifically, we examined: (1) whether CRA exerts therapeutic effects on FLS and in a collagen-induced arthritis (CIA) rat model; (2) whether these effects involve the NF-κB and/or PI3K/AKT signaling pathways; and (3) the relationship between NF-κB and PI3K/AKT signaling in mediating CRA activity. These findings may provide preclinical evidence supporting future development of CRA for RA, potentially including nano-controlled delivery strategies or combination approaches^21^. Limited prior evidence suggests that oral administration of CRA-containing preparations can influence metabolic parameters^22,23^; therefore, our results may also inform the feasibility of oral CRA-based strategies for further translational evaluation in RA.

## Materials and methods

### Isolation, cultivation, and identification of FLS

We used 7-week-old SD (Sprague-Dawley) female rats (Cavens, Changzhou, China) to establish a CIA rat model. The animal experiments were approved by the experimental animal welfare ethics committee of Nanjing Medical University (SYXK (Su) 2018-0020, and we confirm that all experiments were performed in accordance with relevant guidelines and regulations). The steps for establishing the CIA rat model were as follows. We first isolated, cultured, and identified FLS. The rats were anesthetized with 0.6% pentobarbital sodium (40 mg/mL). A mixed emulsion of bovine type Ⅱ collagen and complete Freund’s adjuvant (Chondrex, Redmond, WA, USA) was injected subcutaneously at two points in the tail root of rats for the first immunization. One week later, the same volume of bovine type II collagen and incomplete Freund’s adjuvant emulsion was used for a second immunization as described above. The arthritis score for the CIA rats was evaluated approximately 1 week after the second immunization. When the score was more than 4, synovial tissue was separated from the CIA rat knee joint with a stereoscopic microscope. The synovial tissue was digested and isolated with 0.2% collagenase type II. FLS isolated by digestion were cultured in DMEM high glucose medium (Gibco, Gaithersburg, MD, USA) in a 5% CO_2_ incubator at 37℃. Normal rat FLS were isolated, extracted, and cultured as described previously^24^ (This experiment followed the ARRIVE guidelines 2.0).

We used immunocytochemical staining for the identification of FLS. Detailed methods can be found in our previously published articles^24^ (We confirm that all experimental methods in this study were strictly implemented in accordance with the operating guidelines provided by the reagent company).

### Drug toxicity test of CRA

Experimental animals were divided into three groups: the blank group, the no-drug group, and the CRA group. We cultured FLS at a density of 5 × 10^3^ cells per well in 96-well plates overnight (the blank group did not receive cells, while the no-drug group and the CRA group were inoculated with cells). The CRA group included seven drug concentrations: 0 µg/mL, 1 µg/mL, 2 µg/mL, 4 µg/mL, 6 µg/mL, 8 µg/mL, and 10 µg/mL (MCE, Shanghai, China). Each group was designed with five multiple wells. The next day, the blank group and the no-drug group were replaced with fresh medium, while the CRA group was replaced with medium containing different concentrations of CRA. After incubation for 24 h, 10 µL of CCK-8 solution (Beyotime, Shanghai, China) was added to each well in the dark. After incubation for 2 h, absorbance was measured at 450 nm with a microplate reader (BioTek Epoch, USA). The appropriate concentration of CRA was selected based on results.

### Enzyme linked immunosorbent assay (ELISA)

Cells were divided into four groups: HC-FLS group, CIA-FLS group, CIA-FLS + CRA (6 µg/mL) group, and the CIA-FLS + Indo (4 µg/mL) group (indomethacin, Indo, positive control drug). The four groups of cells were cultured overnight in 6-well plates (2 × 10^5^ cells/well). The HC-FLS group and the CIA-FLS group were not treated with drugs, while the CIA-FLS + CRA group and the CIA-FLS+Indo group were treated with CRA (6 µg/mL) and Indo (4 µg/mL), respectively, for 24 h. Finally, the supernatants of each group of cells were collected. The levels of IL-6, IL-1β, and TNF-α in the supernatants of each group were assessedaccording to the ELISA kit instructions (Multisciences, Zhejiang, China).

### Western blot assay

We extracted total proteins from cells using RIPA buffer (Servicebio, Wuhan, China) containing protease and phosphatase inhibitors (Beyotime, Shanghai, China). Proteins were separated by SDS-PAGE and transferred to nitrocellulose membranes that were blocked with 5% BSA at room temperature for 1 h. Then, primary antibodies reactive with specific proteins (the host species was rabbit): P65, p-P65, IκBα, p-IκBα, PI3K, p-PI3K, AKT, and p-AKT) (ABclonal, Wuhan, China) were added, at the appropriate concentrations, and the membranes incubated at 4 °C overnight. The next day, the membranes were washed three times with TBST. Then, goat anti-rabbit secondary antibody coupled with HRP was incubated at room temperature for 1 h, the membranes were washed three times with TBST, and ECL chemiluminescence (NCM Biotechnology, Suzhou, China) was used to visualize specific protein bands. GAPDH was used as an internal reference.

### Immunofluorescence detection of NF-κB P65 nuclear transport

A nuclear translocation assay kit (Beyotime, Shanghai, China) was used to detect the nuclear transport of NF-κB. Cells were divided into three groups: the HC-FLS group, the CIA-FLS group, and the CIA-FLS + CRA (6 µg/mL) group. The cells were inoculated into three 20 mm glass bottom cell culture dishes, and cultured for 24 h. The cells in the HC-FLS group and the CIA-FLS group were not treated with drugs. The cells in the CIA-FLS + CRA group were treated with CRA for 24 h. Then the cells were placed in a fixative solution for 5–15 min. Next, an immunostaining blocking solution was added and the dishes sealed at room temperature for 1 h. NF-κB P65 specific antibody was added and the dishes incubated overnight at 4 °C. Anti-rabbit Cy3 was added and incubated at room temperature for 1 h. Finally, DAPI was added at room temperature for approximately 5 min, followed by a dropwise addition of an appropriate amount of anti-fluorescence quenching sealing liquid. Cover glasses were sealed and cells observed with a fluorescence microscope (Confocal Microscope ZEISS LSM 900 for Materials, city, Germany). NF-κB staining was red fluorescence, and DAPI staining was blue fluorescence.

### Grouping and evaluation of the therapeutic effect of CRA for CIA rats

CRA or Indo were used to evaluate their therapeutic effect in the CIA model. Twenty-four 7-week-old female rats were randomly divided into four groups (six rats in each group): control group (normal rats), CIA model group, CRA treatment group (oral gavage administration, 10 mg/kg), and Indo positive control group (intra-gastric administration, 1 mg/kg). The concentrations of CRA and Indo by gavage were as described previously^25,26^. CRA and Indo were administered by gavage for 21 days. The control group and CIA model group were given the same volume of normal saline by gavage. Arthritis score and body weight were measured weekly from day 0 of intra-gastric administration. The arthritis scores of rats were as follows: 0, no erythema and swelling; 1, erythema and mild swelling limited to the middle of foot or ankle joint; 2, erythema and moderate swelling from ankle to foot; 3, erythema and swelling from ankle joint to joint; 4, erythema and severe swelling including ankle, foot, and toe.

The rats were weighed 24 h after the last administration. After the rats were anesthetized by intraperitoneal injection of 0.6% pentobarbital sodium (40 mg/mL), the thymus and spleen were quickly removed, rinsed with normal saline, and surfaces dried with gauze. Their mass (wet mass) was weighed and their organ index calculated: organ index = organ wet mass (mg)/rat mass (g).

### Centrifugation of heart blood for ELISA

Twenty-four hours after the rats received the last emulsion administration, 1 mL of blood was collected from the heart. The blood was left at room temperature for 30 min. Then, centrifuged at 1000 × *g* for 10 min at 4 °C. Serum was aspirated and transferred to liquid nitrogen for preservation. The levels of IL-1β, IL-6, and TNF-α in rat serum were assessed according to the instructions of the ELISA kit.

### Histopathologic staining

The ankle joints of the hind limbs of rats were fixed in 10% neutral formaldehyde and sectioned after decalcification, dehydration, and paraffin embedding. The tissue sections were stained as follows: (1) hematoxylin eosin (HE) (Servicebio, Wuhan, China) staining, and (2) safranin O-fast green (Servicebio, Wuhan, China) staining. Staining was performed according to the manufacturer’s instructions, and the pathological changes in ankle tissues were observed with a light microscope (Nikon, Japan). The pathologic changes in cartilage and synovium of the joint were scored according to the standard^27^.

### Micro-computed tomography (micro-CT)

CIA rats were divided into separated into four groups, as described above. A NMC-200 Micro- CT scanner (Pingseng Scientific, KunShan, China) was used to scan right hind limbs of rats at the reconstructed pixel size of 2 µM. Recon software was used for reconstruction and data analysis software, Avatar, to analyze the related parameters of the trabecular region, including bone mineral density (BMD, g/cm^3^), bone volume/total volume fraction (BV/TV, %), trabecular thickness (Tb.Th, µm), and trabecular separation (Tb. Sp, µm). All samples were analyzed in the same area to obtain the required parameter values for data export.

### Statistical analysis

The weight and arthritis score of rats in vivo study were analyzed using ordinary two-way ANOVA, while the remaining data were analyzed using ordinary one-way ANOVA.All data are presented as means ± standard deviation (SD) using GraphPad Prism 8.0.2. A *P* < 0.05 was considered statistically significant.

## Results

### Appropriate concentration selection of CRA and Indo

**Fig. 1:**
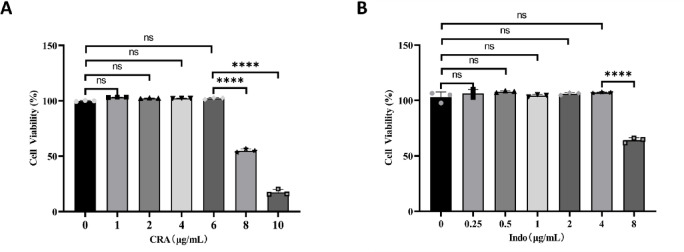
The effects of Corosolic acid (CRA) and indomethacin (Indo) on the viability of normal rats FLS cells. (**A**) FLS cells were treated with different concentrations of CRA solution (0, 1, 2, 4, 6, 8, 10 µg/ml) for 24 h. (**B**) Different concentrations of Indo solution (0, 0.25, 0.5, 1, 2, 4, 8 µg/ml) were used to intervene FLS cells for 24 h. Data are presented as mean ± standard error (mean ± SEM).

When the concentration of CRA was increased to 8 µg/mL, the activity of rat FLS decreased significantly (*P* < 0.0001). Therefore, we selected the concentration of CRA as 6 µg/mL for follow-up experiments (Fig. 1A).

When the concentration of Indo increased to 8 µg/mL, the activity of rat FLS decreased significantly (*P* < 0.0001). Therefore, we selected the concentration of Indo as 4 µg/mL for the follow-up experiments (Fig. 1B).

### ELISA confirmed that CRA inhibits FLS inflammation in CIA rats

**Fig. 2:**
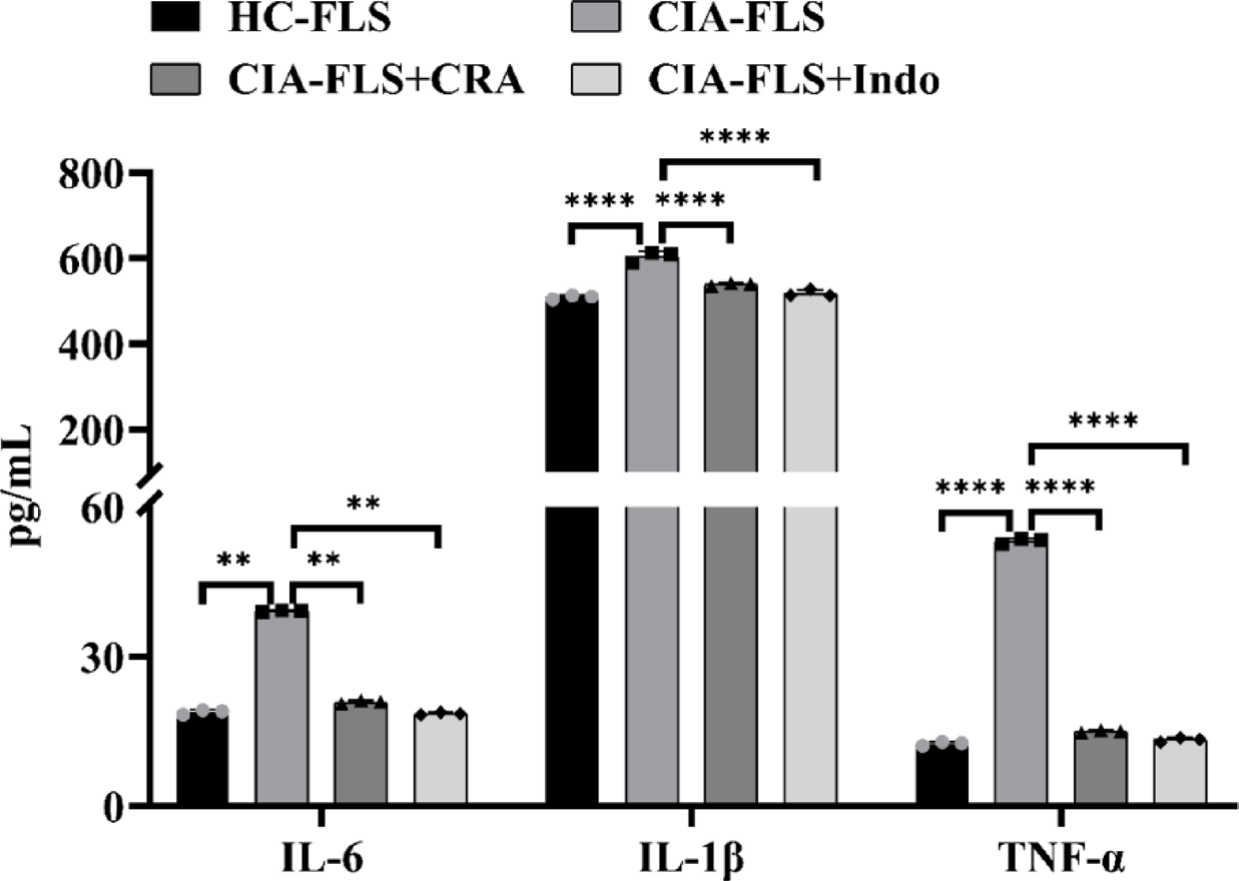
The effects of CRA on the production of IL-6, IL-1β and TNF-α in cell supernatants were detected by ELISA. Data are presented as mean ± SEM.

The concentration of inflammatory factors in the CIA-FLS group was significantly higher than that in the HC-FLS group (*P* < 0.01). Compared with the CIA-FLS group, the concentration of inflammatory factors in the CIA-FLS + CRA group and the positive control, the CIA-FLS+Indo group, were significantly decreased (*P* < 0.01) (Fig. 2). Therefore, CRA significantly inhibited the inflammation of CIA-FLS.

### Effect of CRA on NF-κB and PI3K/AKT signaling pathways

**Fig. 3:**
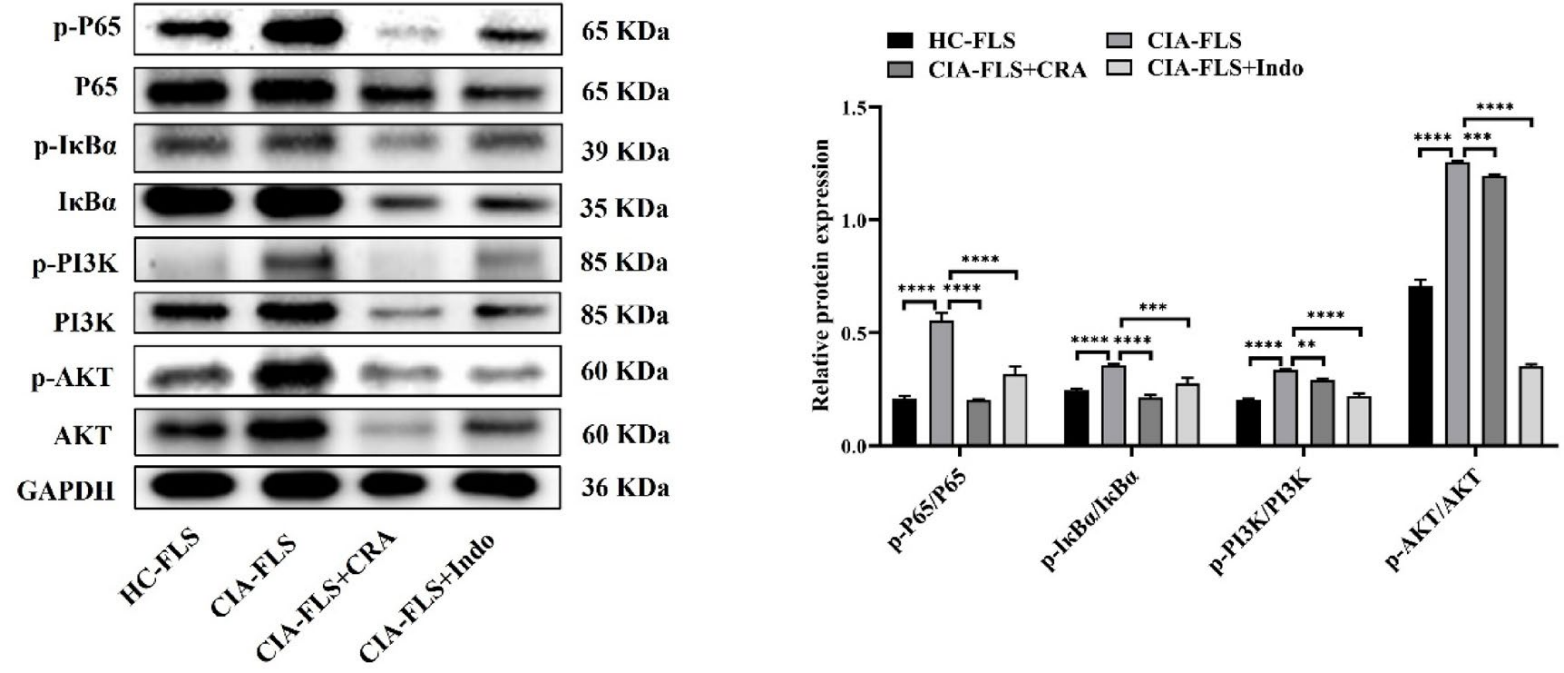
CRA significantly inhibited the phosphorylation levels of the pathway of CIA-FLS. The effect of CRA on NF-κB/PI3K/AKT signaling pathway was detected by Western blot. CRA significantly inhibited the phosphorylation levels of p-P65, p-IκBα, p-PI3K and p-Akt. Data are presented as mean ± SEM.

The phosphorylation levels of the NF-κB and the PI3K/AKT pathways in the CIA-FLS group were significantly higher than those in the HC-FLS group (*P* < 0.0001). Compared with the CIA-FLS group, the phosphorylation levels of the NF-κB and the PI3K/AKT pathways in the CRA intervention group and Indo positive control group were significantly decreased (*P* < 0.01) (Fig. 3). These results indicated that CRA exerted RA anti-inflammatory therapeutic effects by inhibiting the NF-κB and the PI3K/AKT pathways.

**Fig. 4:**
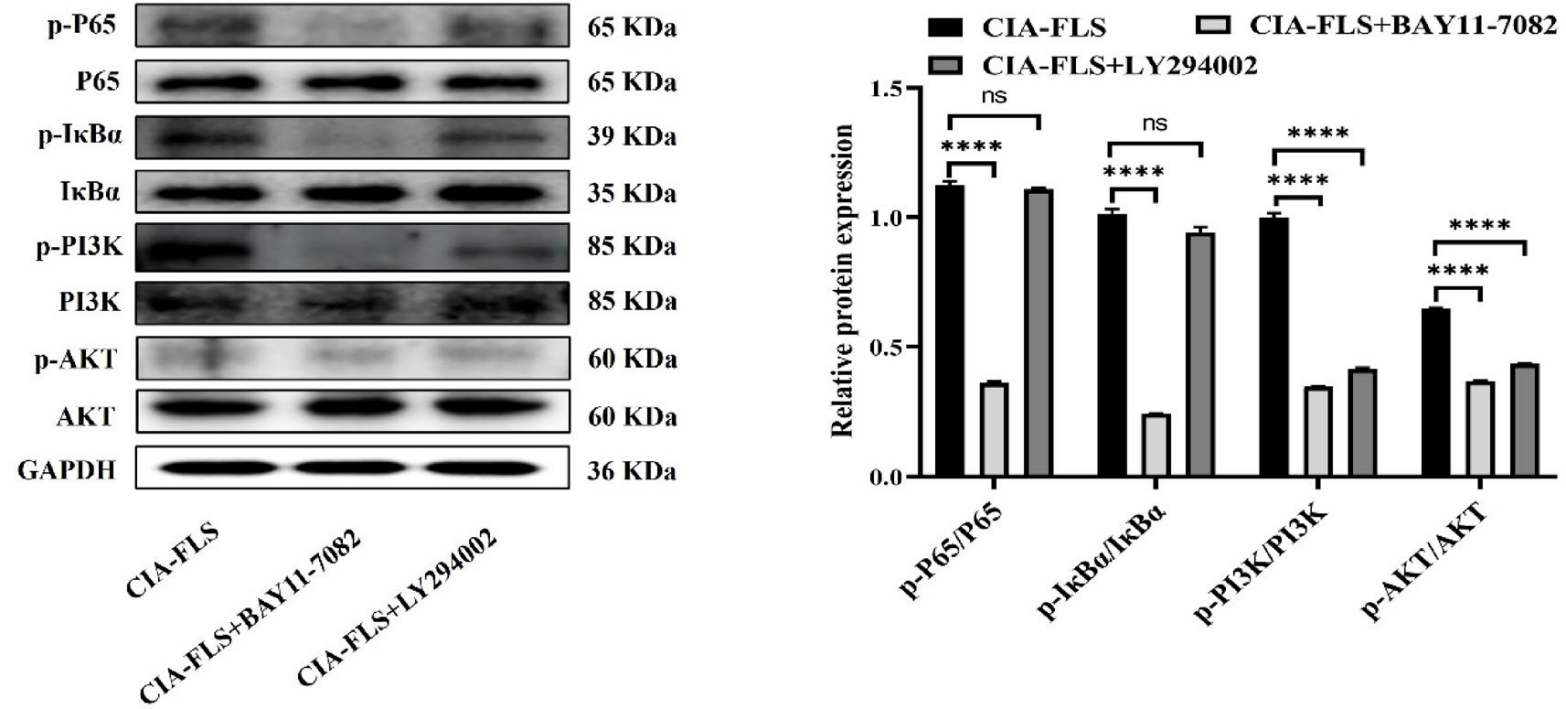
P65 inhibitor (BAY11-7082) and PI3K inhibitor (LY294002) were used to explore the upstream and downstream relationship between NF-κB and PI3K/AKT pathway. The effects of these inhibitors on NF-κB/PI3K/AKT signaling pathway were detected by Western blot. P65 inhibitor significantly decreased the phosphorylation levels of P65 and IκBα, and the phosphorylation levels of PI3K and Akt, key factors of PI3K/AKT signaling pathway, were also significantly decreased. PI3K inhibitors significantly decreased the phosphorylation levels of PI3K and Akt, but the phosphorylation levels of P65 and IκB did not change significantly. Suggesting that NF-κ B is upstream of PI3K/AKT signaling. Data are presented as mean ± SEM.

Compared with the CIA-FLS group, the phosphorylation levels of the key factors of the NF-κB signaling pathway, P65 and IκBα, were significantly reduced in the P65 inhibitor (Bay11-7082) group (*P* < 0.0001). Furthermore, the phosphorylation levels of key factors of the PI3K/AKT signaling pathway, PI3K and AKT, were also significantly reduced (*P* < 0.0001). Compared with the CIA-FLS group, the PI3K inhibitor (LY294002) group showed a significant decrease in the phosphorylation levels of PI3K and AKT (*P* < 0.0001). However, no significant change in the phosphorylation levels of P65 and IκBα was found in this group (Fig. 4). These results indicated that the NF-κB signaling pathway was upstream of the PI3K signaling pathway.

### Effect of CRA on NF-κB P65 nuclear transport

**Fig. 5:**
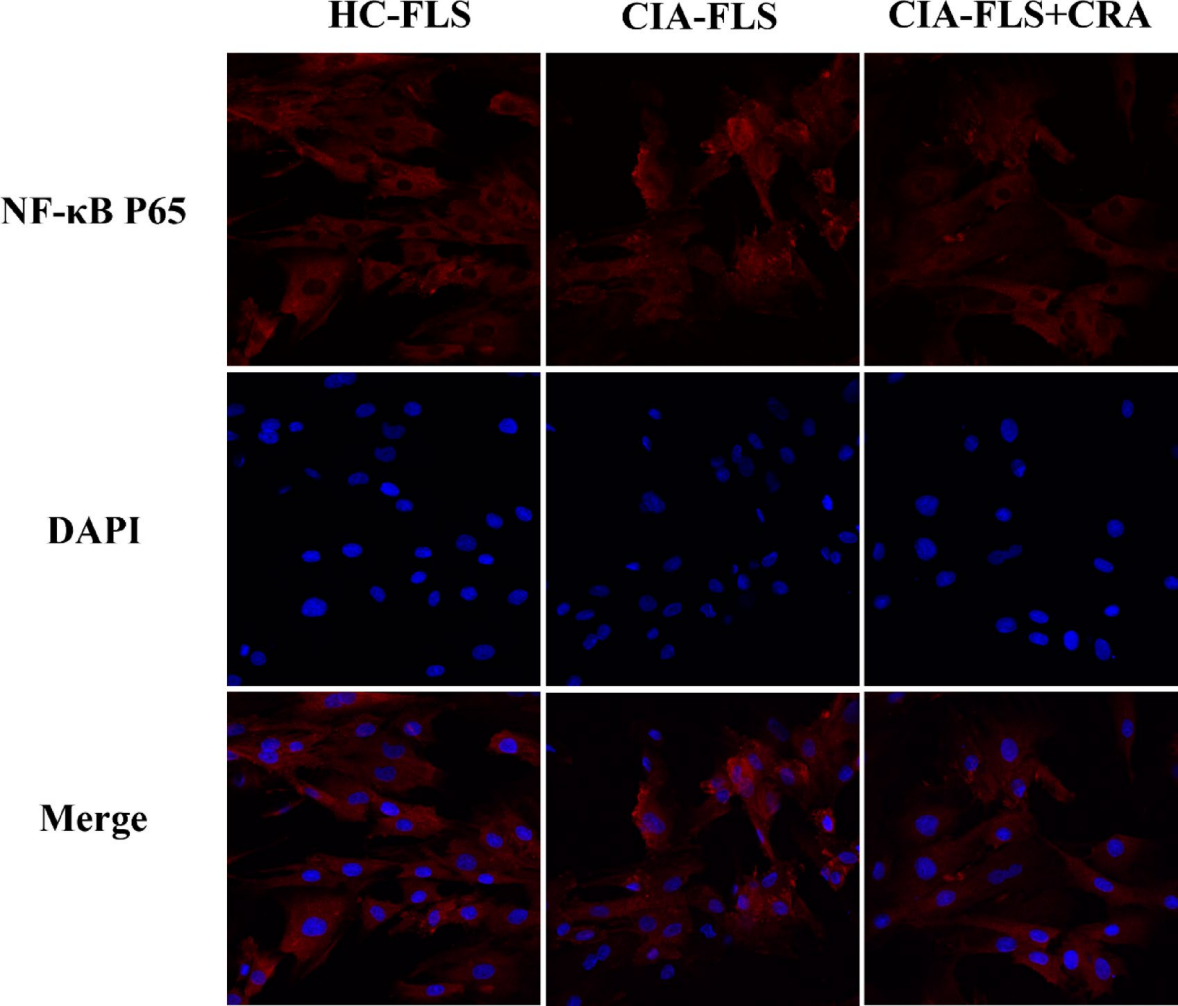
Effects of CRA on NF-κB activation of CIA-FLS. The nuclear translocation of P65 was detected by immunofluorescence combined with DAPI nuclear staining (400×).

We used immunofluorescence to detect the nuclear translocation of NF-κB p65. The results showed that compared with the HC-FLS group, the phosphorylation of NF-κB P65 in the CIA-FLS group transferred readily from the cytoplasm to the nucleus. Therefore, after CRA treatment of CIA-FLS, there was an RA anti-inflammatory effect through dephosphorylation of P65 and transfer from the nucleus to the cytoplasm (Fig. 5).

### Intra-gastric administration of CRA significantly eased the arthritis of CIA rats

**Fig. 6:**
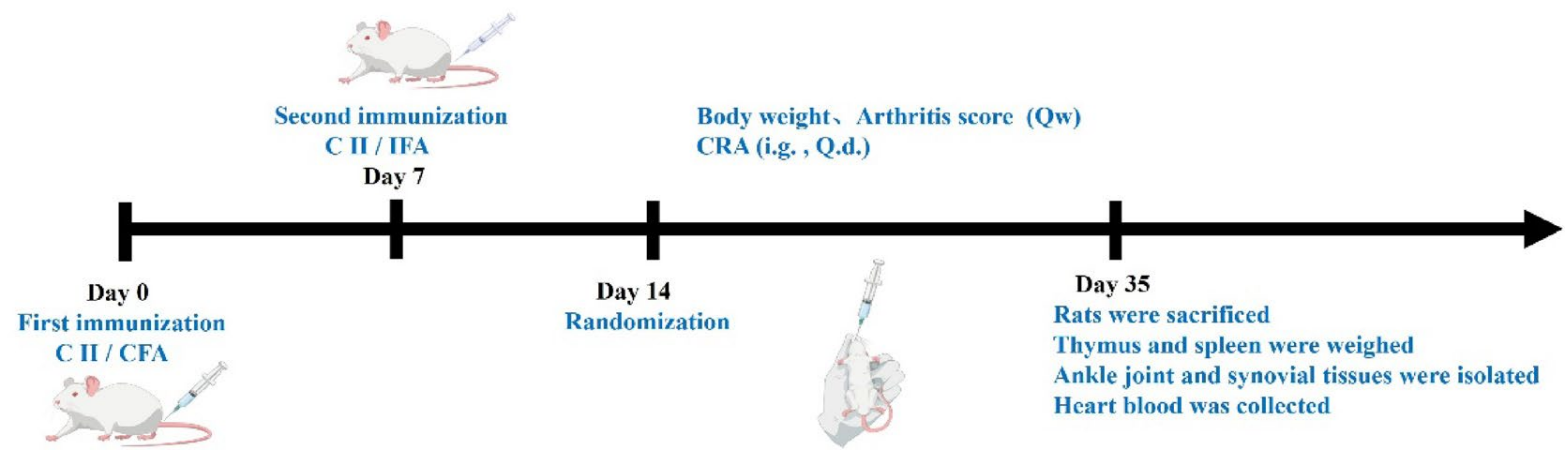
Animal experimental roadmap. (CII: bovine type II collagen; CFA: complete Freund’s adjuvant; IFA: incomplete Freund’s adjuvant. The mixed emulsion of CⅡ and CFA was injected for the first immunization, and one week later, the mixed emulsion of CⅡ and IFA was injected to strengthen the immunization. One week after successful CIA modeling, normal saline, CRA and Indo was administered by gavage once a day for 21 days).

Rats were injected for the first time with equal volumes of CII and CFA emulsion. One week later, the rats were re-injected with equal volumes of CII and IFA emulsion. After successful CIA modeling, rats in the CRA intervention group and Indo positive control group were gavaged with CRA or Indo once a day for 21 days. During this period, rats were scored for arthritis and their body weight measured weekly. After the final intra-gastric administration, rats were sacrificed, thymus and spleen of each rat weighed, ankle joint, and synovial tissues separated, and heart blood collected (Fig. 6).

**Fig. 7:**
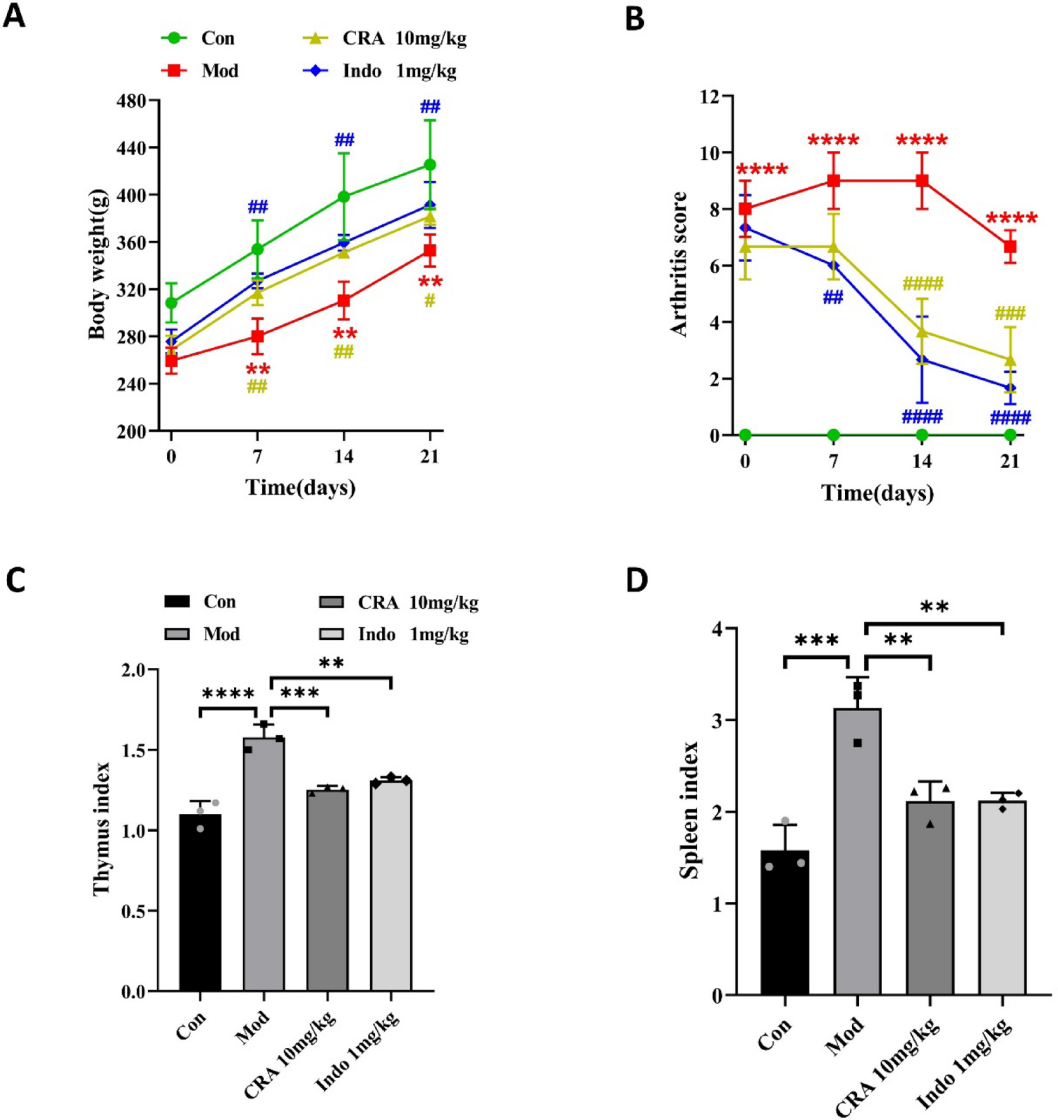
CRA inhibits the development of arthritis in CIA rats. (**A**, **B**) Effects of CRA on body weight and arthritis score of CIA rats at different time points. After CRA and Indo treatment, the body weight of rats significantly increased on the 7th, 14th and 21st days after gavage. The arthritis score of rats began to decrease significantly one week after gavage. (**C**, **D**) The weight changes of thymus and spleen of CIA rats after gavage were shown. After CRA and Indo treatment, the organ index of rats decreased significantly. *Compared with Control (CON) group; #Compared with Model (Mod) group. Data are presented as mean ± SEM.

After the CIA rat model was successfully established, we evaluated the anti-inflammatory effect of CRA on CIA rats. The weight and arthritis scores of rats were assessed once a week from day 0 of intra-gastric administration. The thymus and spleen weight were recorded 24 h after the last administration. During the observation period, the body weight of normal control group rats gradually increased. However, the body weight of the rats in the CIA model group gradually decreased and was significantly different from the control group 1 week after intra-gastric administration (*P* < 0.01). Compared with the CIA model rats, the body weight of rats in the CRA treatment group and Indo positive control group increased significantly on the 7th, 14th, and 21st days after intra-gastric administration (*P* < 0.05) (Fig. 7A).

Compared with the normal control group, the arthritis score of rats in the CIA model group was significantly increased (*P* < 0.0001). Compared with the CIA model group, the arthritis scores of rats in the CRA treatment group and Indo positive controlgroup began to decrease significantly 2 week after gavage (*P* < 0.0001). These results indicated that CRA improved the inflammatory symptoms of CIA rats (Fig. 7B).

Compared with the normal control group, the organ index (thymus and spleen) of rats in the CIA model group was significantly increased (*P* < 0.001). However, compared with the CIA model group, the organ index (thymus and spleen) of rats in the CRA treatment group and the Indo positive control group decreased significantly (*P* < 0.01) (Fig. 7C,D).

### Inflammatory factor analysis of serum

**Fig. 8:**
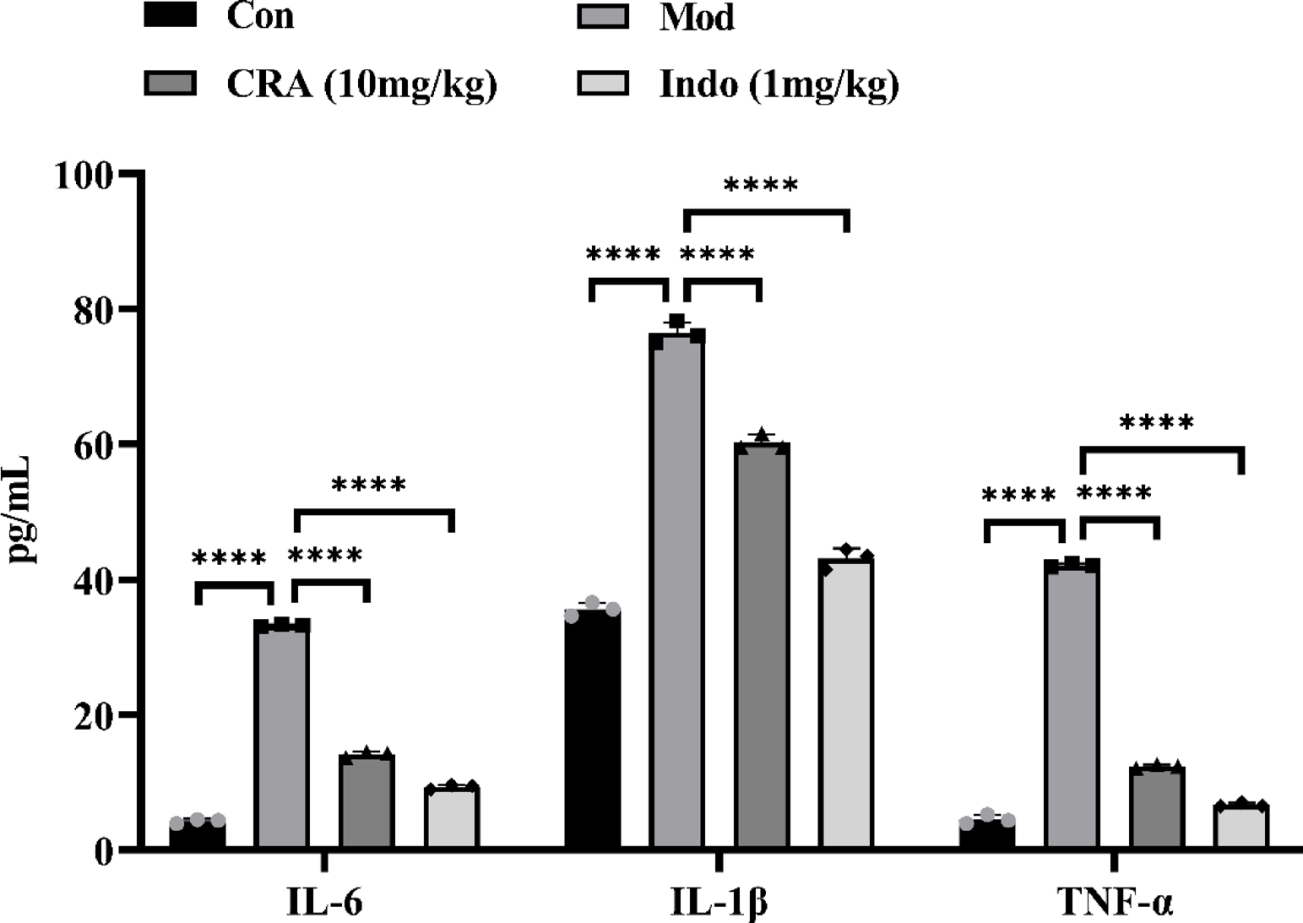
ELISA was used to detect the levels of IL-6, IL-1β and TNF-α in serum of rats. Data are presented as mean ± SEM.

Compared with the normal control group, the concentration of IL-6, IL-1β, and TNF-α in the serum of CIA model group rats was significantly increased (*P* < 0.0001). Compared with the CIA model group, the concentration of IL-6, IL-1β, and TNF-α in the serum of rats in the CRA treatment group and the Indo positive control group was significantly decreased (*P* < 0.0001) (Fig. 8).

### Effect of CRA on the ankle joints of CIA rat: histopathologic results

**Fig. 9:**
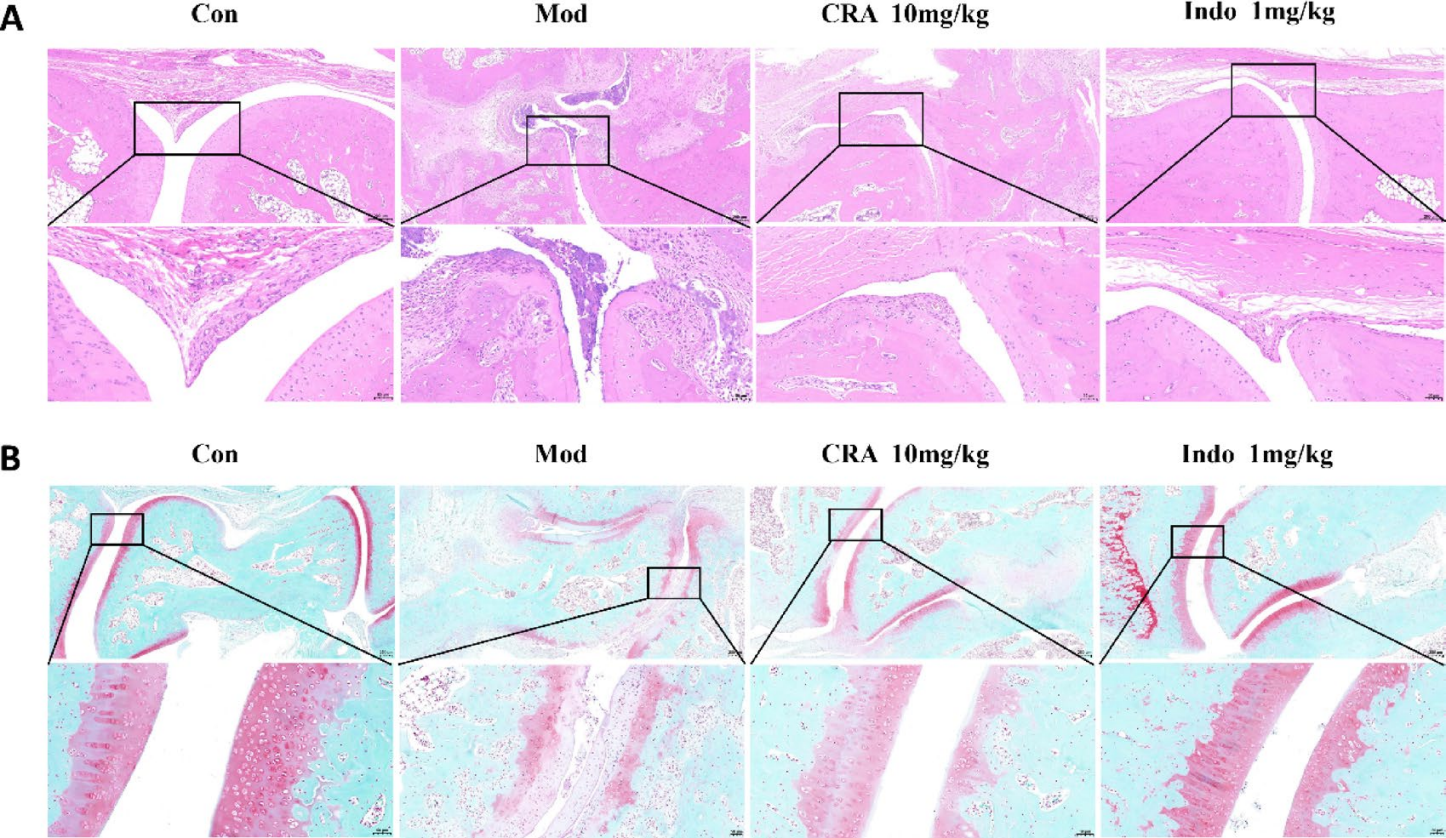
Histological analysis of CIA rats was evaluated by HE staining (**A**) and safranin O-fast green staining (**B**). After treatment with CRA and Indo, the thickness of synovial epithelium decreased and the infiltration of inflammatory cells was inhibited, which also improved cartilage damage (scale bar 50 μm).

Histopathologic results of synovial tissue showed the ankle joints of the normal control group of rats to be thin and soft, with uniform thickness. In contrast, the synovial tissue of the ankle joints of the CIA model group of rats was thickened with inflammatory cell infiltration. Compared with the CIA model group of rats, the CRA treatment group and Indo positive control group of rats had significantly reduced synovial tissue proliferation within the ankle joint, as well as reduced infiltration of inflammatory cells (Fig. 9A).

Sections of ankle joints from normal rats and CIA rats were stained with safranin O-fast green, with cartilage appearing red and bone tissue appearing green. The joint cavity structure of the ankle joint in the normal control group of rats was intact, with a closed cavity, uniform joint space, no abnormal expansion or narrowing, and a smooth cartilage surface. Compared with the normal control group, the joint cavity of the ankle joint in the CIA model group of rats narrowed due to cartilage destruction and synovial hyperplasia, with a cartilage surface that was rough and fibrotic. However, compared with the CIA model group, the CRA treatment group and the Indo positive control group of rats had significantly reduced inflammation, cellular infiltration, and cartilage damage in rat ankle joints. (Fig. 9B).

### Effect of CRA on ankle joint bone invasion in CIA rats

**Fig. 10:**
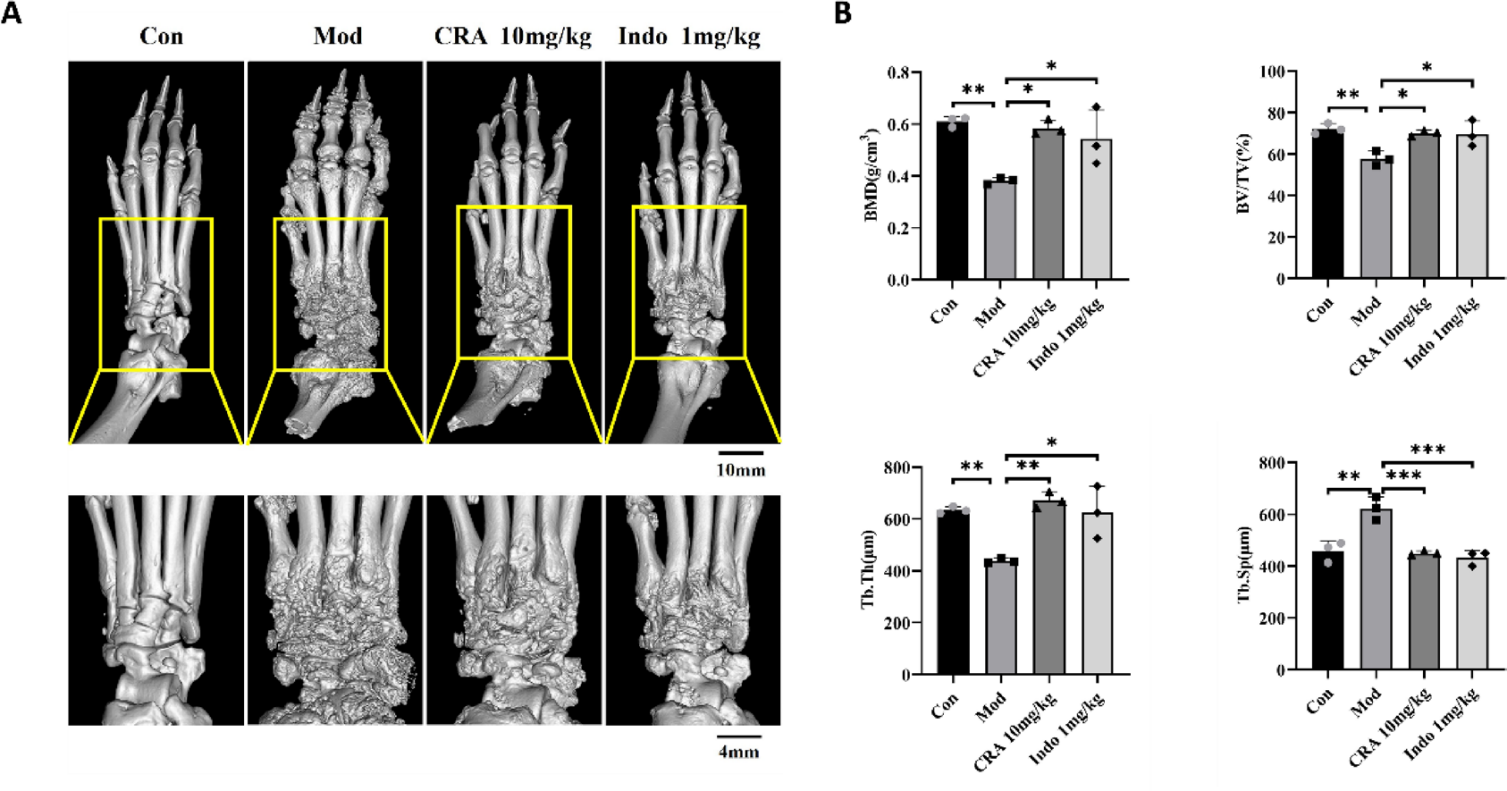
Representative micro CT 3D reconstruction images of CIA rats after 21 days of gavage (front view). (**A**) The bone erosion degree of the right ankle joint of the rat can be seen in the enlarged image. (**B**) Quantitative analysis of bone parameters of the right hind paw of rats in each group (BMD, BV/TV, Tb.Th and Tb.Sp). After CRA and Indo treatment, bone parameters including BMD, BV/TV and Tb.Th were significantly increased, while Tb.Sp was significantly decreased. Data are presented as mean ± SEM.

We used Micro-CT analysis technology to evaluate the effect of CRA on the bone structure and bone mineral density of ankle joint in CIA rats. The three-dimensional (3D) reconstruction image of the right hind paw bone of the rats showed that the ankle joint of the CIA model group rats suffered serious bone damage, with irregular bone defects or depressions of the ankle joint surfaces (Fig. 10A). Compared with the CIA model group of rats, the area of bone erosion in the ankle joint of rats treated with CRA was reduced, new bone tissue was increased, and bone destruction was slowed. Integrity and smoothness were significantly enhanced in the ankle joint of CIA rats treated with Indo. Compared with the normal control group of rats, bone parameters including BMD, BV/TV, and Tb.Th for the CIA model group of rats were significantly decreased (*P* < 0.01). However, compared with the CIA model group of rats, the bone parameters (BMD, BV/TV and Tb.Th) of the CRA treatment group and Indo positive control group of rats were significantly increased (*P* < 0.05). We compared the expression of trabecular spacing (Tb.Sp) in the ankle joint of CIA rats by quantitative analysis. The results showed that compared with the normal control group, Tb.Sp of the CIA model group of rats was significantly increased (*P* < 0.01). However, compared with the CIA model group, Tb.Sp of the CRA treatment group and Indo positive control group of rats decreased significantly (*P* < 0.001) (Fig. 10B). These data indicated that treatment with CRA alleviated ankle joint bone injury of CIA rats, thus preventing further destruction of ankle joint tissue, suggesting that CRA had a protective effect on bone loss.

## Discussion

RA is a chronic autoimmune inflammatory disease in which synovitis and pannus formation are hallmark pathological features. FLS are mesenchymal-derived stromal cells and a major constituent of synovial tissue. Aberrant activation and hyperplasia of FLS contribute substantially to RA pathogenesis. Activated FLS secrete inflammatory cytokines and chemokines that amplify synovial inflammation and promote joint destruction^28^. In the present study, we evaluated the effects of CRA on CIA-FLS and in CIA rats. Our results indicate that CRA exerts anti-inflammatory effects, at least in part, through inhibition of the NF-κB and PI3K/AKT signaling pathways.

Clinically, RA treatment is largely based on NSAIDs, DMARDs, and biologic agents, which aim to reduce inflammation and pain and to slow disease progression. However, long-term therapy can lead to adverse effects (e.g., gastrointestinal symptoms, hepatic and renal injury, osteoporosis, or infection risk), and some agents are costly or show limited efficacy in subsets of patients^29,30^. Natural triterpenoids have been investigated because of their anti-inflammatory properties and reported efficacy in arthritis models, often with limited cytotoxicity^13,31,32^. CRA is a pentacyclic triterpenoid^33^ with reported anti-inflammatory and metabolic regulatory activities^34^. For example, CRA has been reported to ameliorate high-fat diet- and carbon tetrachloride–induced nonalcoholic steatohepatitis by modulating TGF-β1/Smad2, NF-κB, and AMPK signalingpathways^25^, and to suppress acute inflammation by inhibiting IRAK-1 phosphorylation in macrophages^35^. In addition, CRA activates autophagy via the PI3K/AKT/mTOR pathway and protects chondrocytes from IL-1β-induced extracellular matrix degradation in osteoarthritis models^18^. A loquat leaf extract containing CRA has also been reported to reduce paw swelling in the complete Freund’s adjuvant arthritis model^14^. Nevertheless, the effects of purified CRA on RA have not been previously reported.

In our in vitro experiments, CRA reduced inflammatory mediator levels in CIA-FLS culture supernatants, supporting an anti-inflammatory effect. We selected Indo as a positive control based on its established anti-inflammatory activity and prior use in related experimental settings^26,36^. Mechanistically, CRA decreased the phosphorylation of key proteins in the NF-κB and PI3K/AKT pathways, including p65, IκBα, PI3K, and AKT. Both pathways are well recognized contributors to inflammatory signaling and RA pathogenesis^37,38^. To explore potential pathway crosstalk, we used pharmacological inhibitors. Inhibition of NF-κB signaling with the p65 inhibitor BAY 11-7082 suppressed both NF-κB activation and PI3K/AKT signaling, whereas PI3K inhibition with LY294002 attenuated PI3K/AKT signaling without significantly affecting NF-κB activation. These findings suggest that, in CIA-FLS, NF-κB may function upstream of PI3K/AKT, and that CRA may exert its effects by inhibiting this NF-κB–PI3K/AKT axis. Because pharmacological inhibitors can have off-target effects, further validation using genetic approaches (e.g., siRNA knockdown or overexpression/rescue experiments) would strengthen causal inference.

In vivo, CRA administration by oral gavage significantly reduced the arthritis index and organ indices in CIA rats and significantly increased body weight relative to untreated CIA animals. CRA also reduced serum levels of inflammatory cytokines. Micro-CT analysis and histopathological evaluation of ankle joints further supported that CRA mitigated bone erosion and synovial hyperplasia in CIA rats. Collectively, these data indicate that CRA has therapeutic potential in this RA model.

This study has several limitations. First, the pathogenesis of RA involves multiple interconnected signaling pathways, whereas the present study focused primarily on the NF-κB and PI3K/AKT signaling axes. Second, the experimental analyses were conducted using FLS derived from CIA rats, rather than human RA synovial tissue specimens. Future studies should incorporate validation using clinical RA tissue samples and perform broader signaling pathway profiling. Third, this study provides preliminary evidence of the short-term therapeutic effects of CRA, however, its bioavailability limitations and potential off-target binding risks warrant caution prior to clinical translation. Fourth, based on the current evidence, we recommend that the use of CRA be strictly confined to regulated preclinical research and mechanistic exploration. Future investigations should focus on: (1) improving pharmacokinetic profiles through novel drug delivery systems while concurrently assessing safety; and (2) conducting long-term toxicological evaluations in compliance with Good Laboratory Practice (GLP) standards to further establish its druggability potential.

## Conclusion

Our study demonstrates that CRA exerts therapeutic effects in CIA rats in vivo and suppresses inflammatory cytokine production by CIA-FLS in vitro. Mechanistically, CRA inhibits NF-κB and PI3K/AKT signaling, and our inhibitor experiments suggest that NF-κB may act upstream of PI3K/AKT in this context. Further elucidation of CRA’s mechanism of action will provide preclinical rationale for evaluating CRA as a potential therapeutic candidate for RA.

## Supplementary Information

Below is the link to the electronic supplementary material.

Supplementary Material 1

Supplementary Material 2

## Data Availability

Data will be made available on request.
