# Supplementary material for: Corosolic acid alleviates rheumatoid arthritis by down regulation of the NF-κB/PI3K/AKT signaling pathway
Source: Sci Rep. 2026 Mar 28;16:10760. doi: 10.1038/s41598-026-46070-3 (PMC13040055; doi:10.1038/s41598-026-46070-3)
Supplement: Supplementary file 1 — Supplementary Material 1 [file 41598_2026_46070_MOESM1_ESM.pptx]

## Slide 1
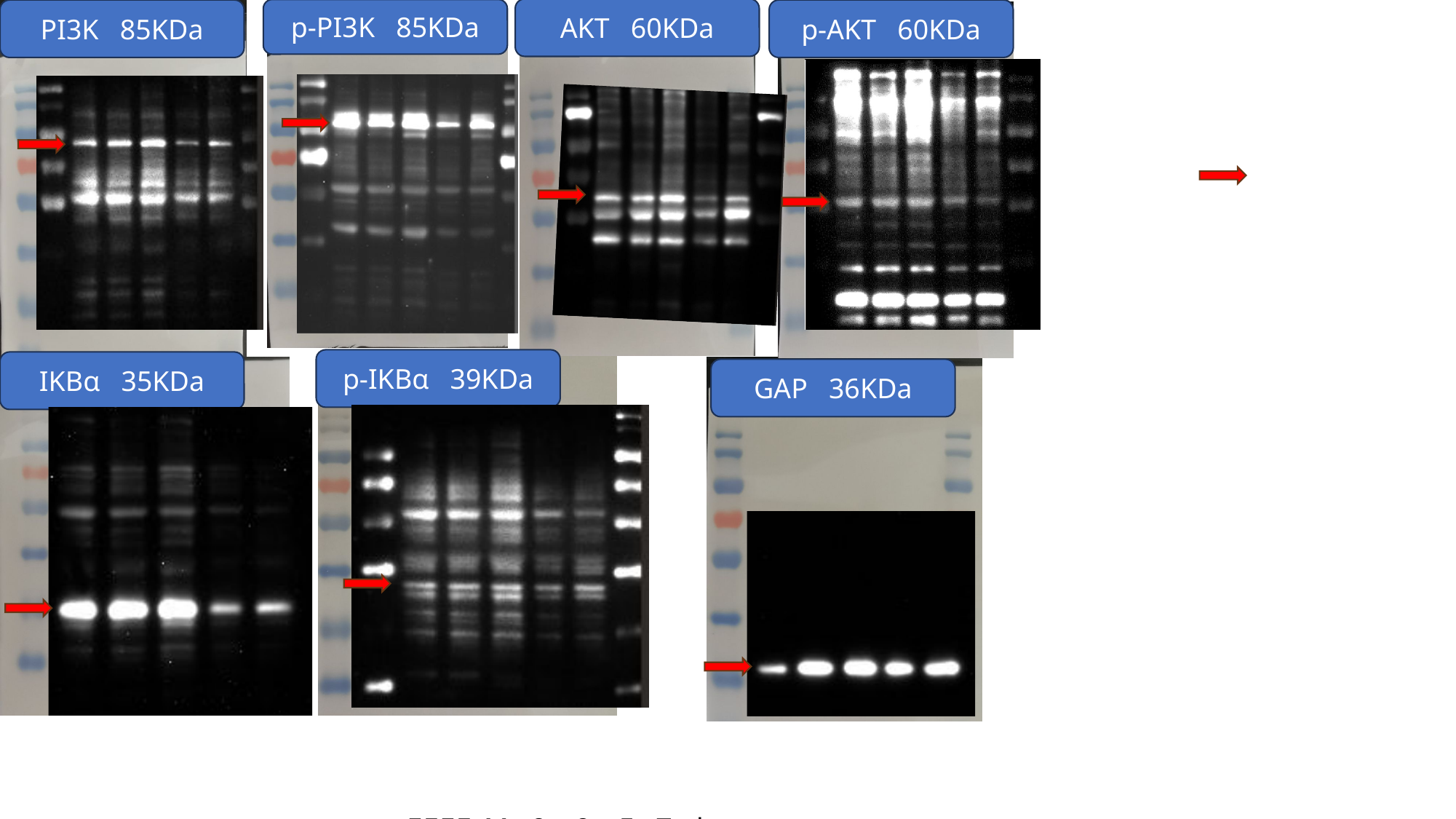

PI3K 85KDa
p-AKT 60KDa
AKT 60KDa
p-PI3K 85KDa
p-IKBα 39KDa
IKBα 35KDa
GAP 36KDa
上样量：11 9 9 5 7 ul
蛋白：Rat-FLS HC HC+LPS CIA+LPS +科罗 +吲哚

## Slide 2
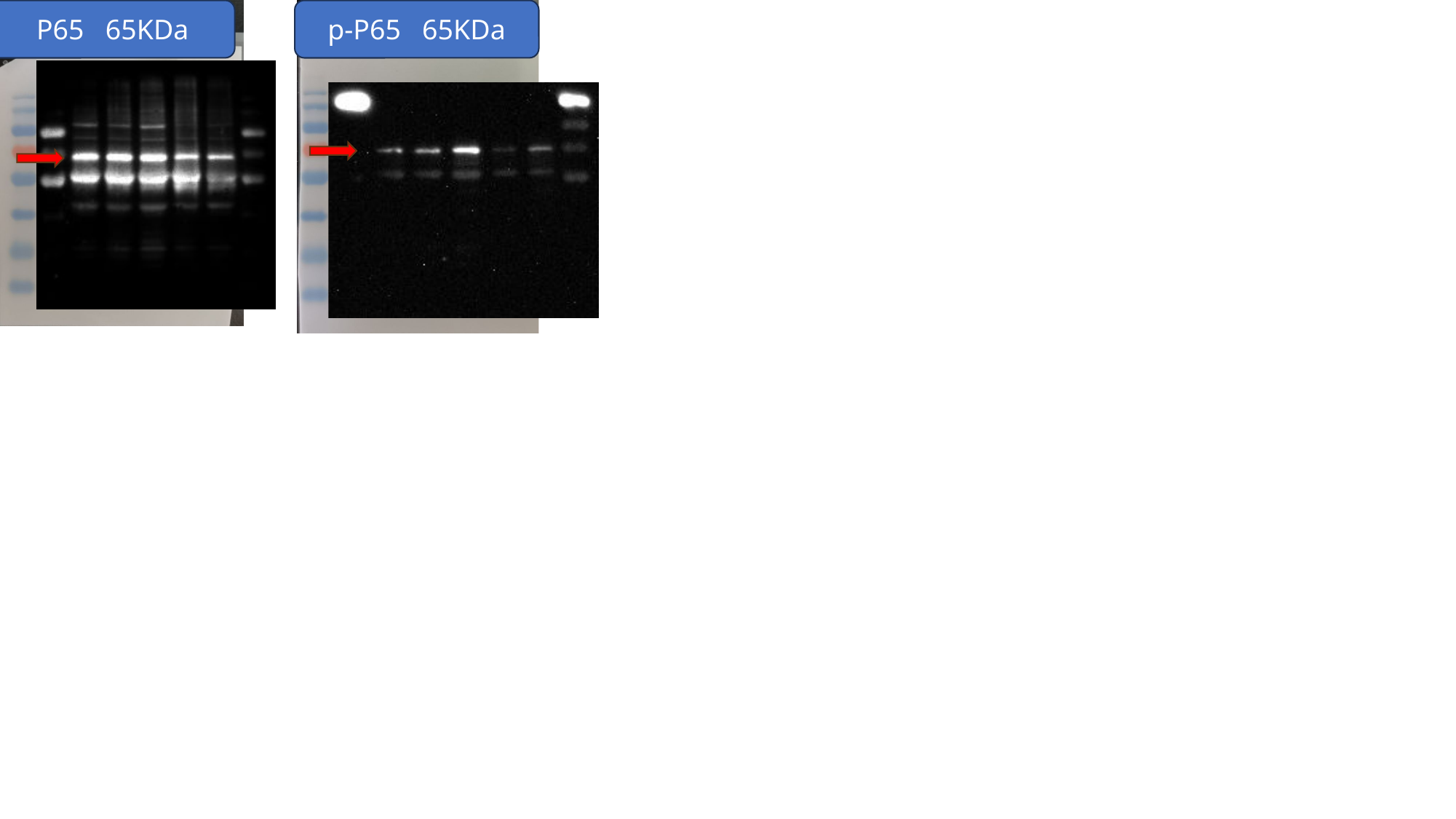

P65 65KDa
p-P65 65KDa

## Slide 3
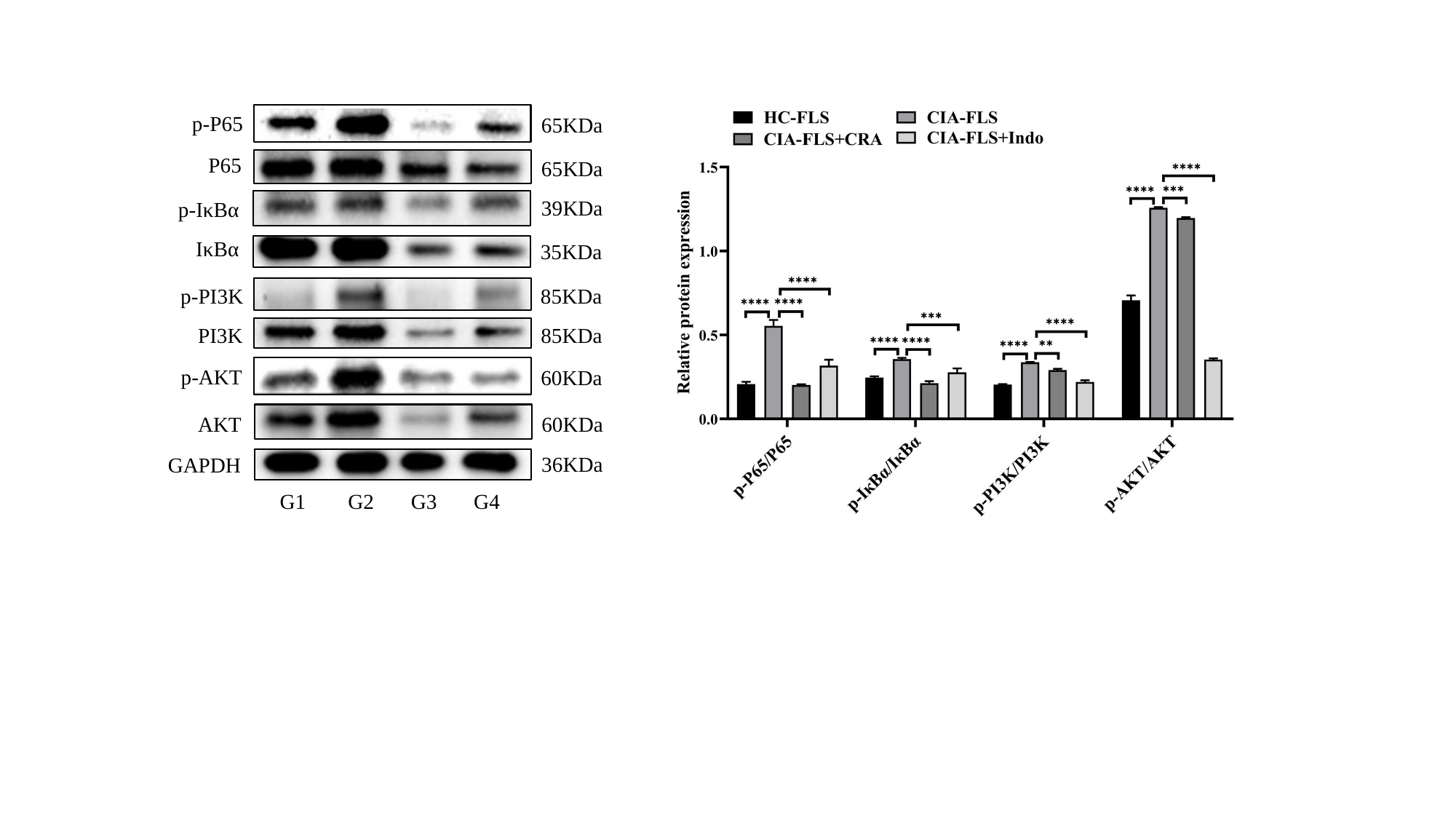

p-P65
65KDa
P65
65KDa
39KDa
p-IκBα
IκBα
35KDa
85KDa
p-PI3K
PI3K
85KDa
p-AKT
60KDa
60KDa
AKT
36KDa
GAPDH
 G1 G2 G3 G4
