# Supplementary material for: Corosolic acid alleviates rheumatoid arthritis by down regulation of the NF-κB/PI3K/AKT signaling pathway
Source: Sci Rep. 2026 Mar 28;16:10760. doi: 10.1038/s41598-026-46070-3 (PMC13040055; doi:10.1038/s41598-026-46070-3)
Supplement: Supplementary file 2 — Supplementary Material 2 [file 41598_2026_46070_MOESM2_ESM.pptx]

## Slide 1
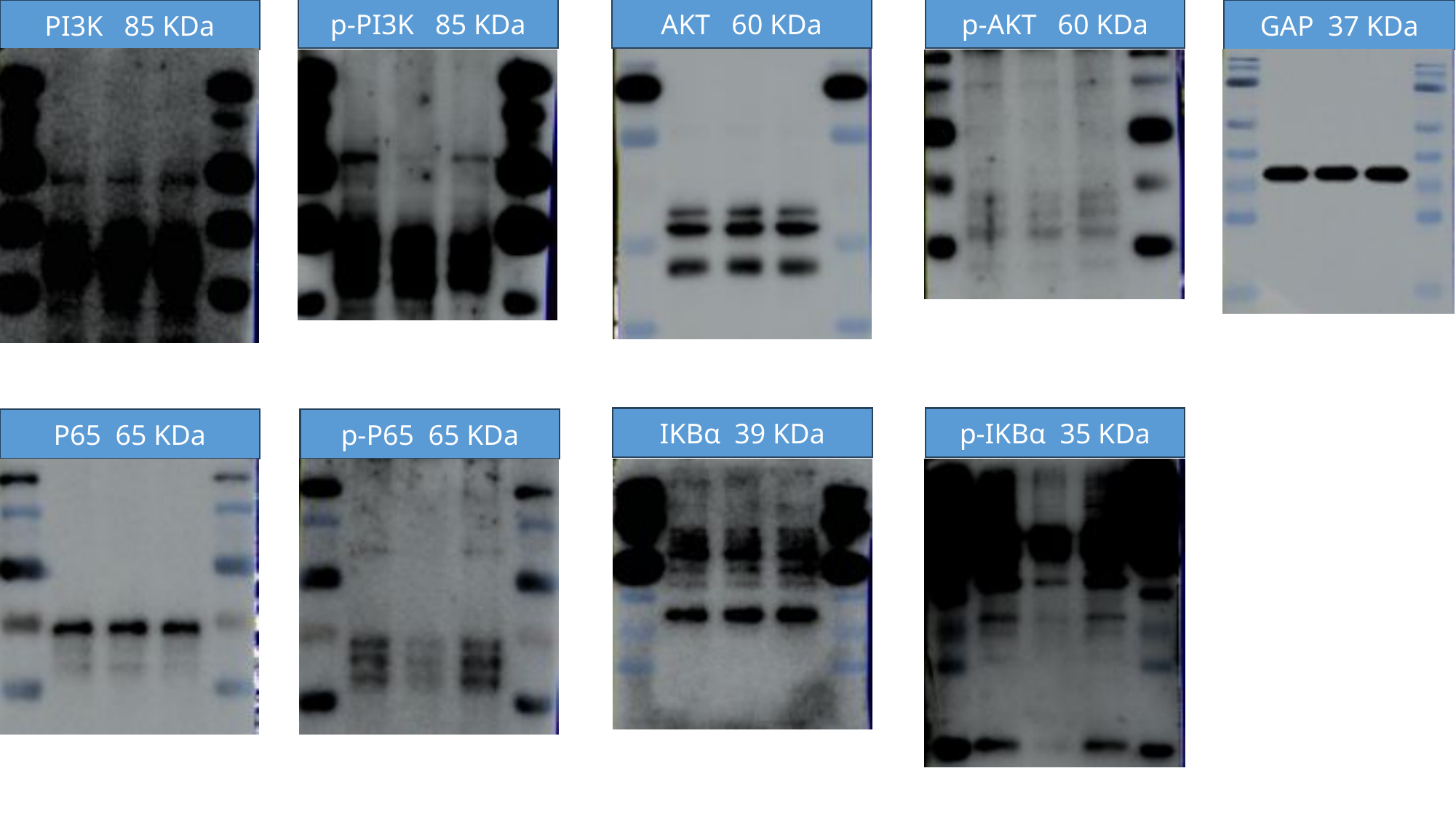

p-PI3K 85 KDa
AKT 60 KDa
p-AKT 60 KDa
PI3K 85 KDa
GAP 37 KDa
IKBα 39 KDa
p-IKBα 35 KDa
P65 65 KDa
p-P65 65 KDa

## Slide 2
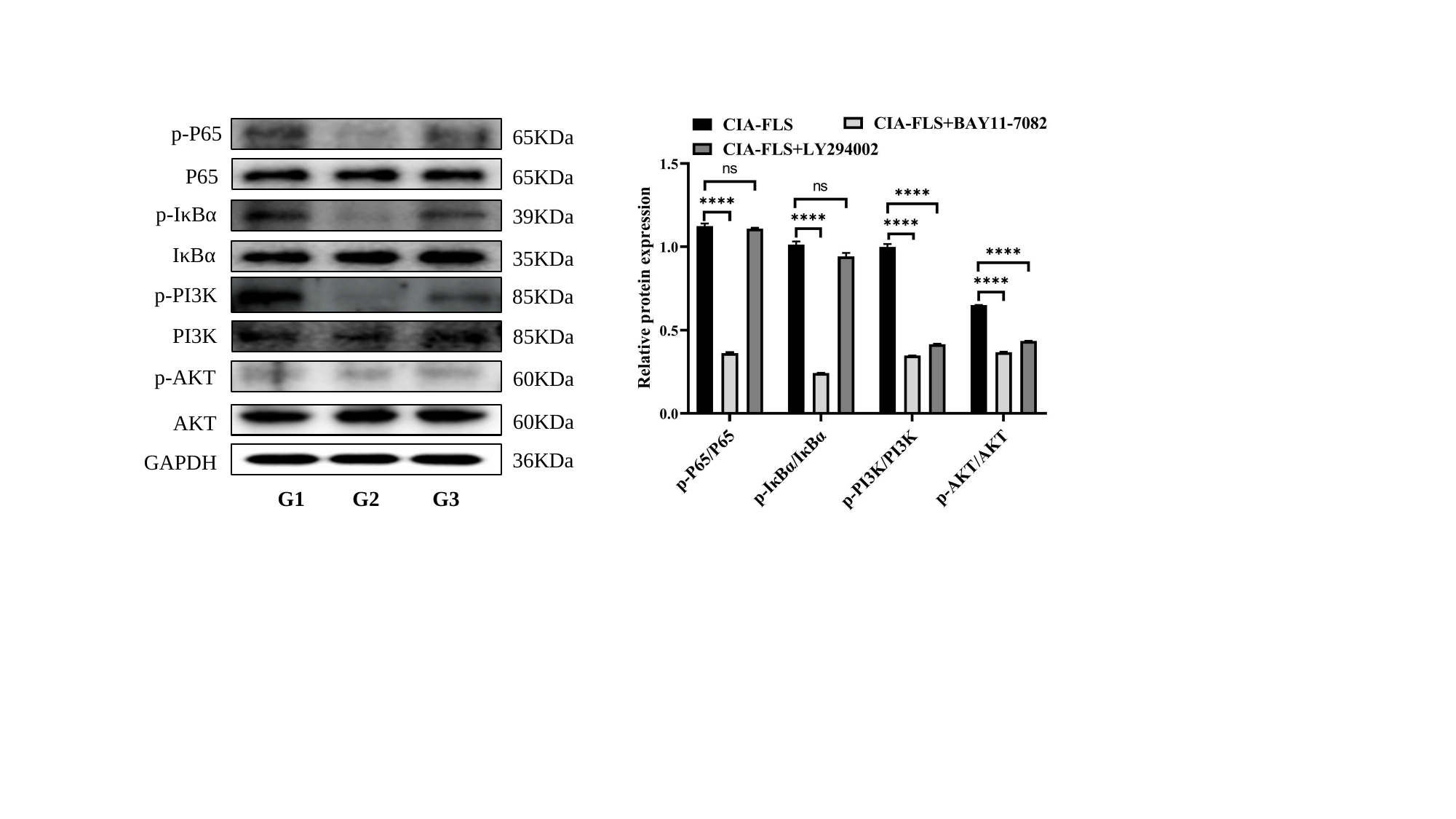

p-P65
65KDa
P65
65KDa
p-IκBα
39KDa
IκBα
35KDa
p-PI3K
85KDa
PI3K
85KDa
p-AKT
60KDa
60KDa
AKT
36KDa
GAPDH
 G1 G2 G3
